# Automatic Structuring of Ontology Terms Based on Lexical Granularity and Machine Learning: Algorithm Development and Validation

**DOI:** 10.2196/22333

**Published:** 2020-11-25

**Authors:** Lingyun Luo, Jingtao Feng, Huijun Yu, Jiaolong Wang

**Affiliations:** 1 School of Computer Science University of South China Hengyang China; 2 Hunan Medical Big Data International Science and Technology Innovation Cooperation Base Hengyang China; 3 Clinical Laboratory Medicine Center Shenzhen Hospital Southern Medical University Shenzhen China

**Keywords:** ontology, automatic structuring, Foundational Model of Anatomy, lexical granularity, machine learning

## Abstract

**Background:**

As the manual creation and maintenance of biomedical ontologies are labor-intensive, automatic aids are desirable in the lifecycle of ontology development.

**Objective:**

Provided with a set of concept names in the Foundational Model of Anatomy (FMA), we propose an innovative method for automatically generating the taxonomy and the partonomy structures among them, respectively.

**Methods:**

Our approach comprises 2 main tasks: The first task is predicting the direct relation between 2 given concept names by utilizing word embedding methods and training 2 machine learning models, Convolutional Neural Networks (CNN) and Bidirectional Long Short-term Memory Networks (Bi-LSTM). The second task is the introduction of an original granularity-based method to identify the semantic structures among a group of given concept names by leveraging these trained models.

**Results:**

Results show that both CNN and Bi-LSTM perform well on the first task, with F1 measures above 0.91. For the second task, our approach achieves an average F1 measure of 0.79 on 100 case studies in the FMA using Bi-LSTM, which outperforms the primitive pairwise-based method.

**Conclusions:**

We have investigated an automatic way of predicting a hierarchical relationship between 2 concept names; based on this, we have further invented a methodology to structure a group of concept names automatically. This study is an initial investigation that will shed light on further work on the automatic creation and enrichment of biomedical ontologies.

## Introduction

### Background

Biomedical ontologies are formalized representations of concepts and the relationships among these concepts for the biomedical domain, and they play a vital role in many medical settings [1]. The constructions of ontologies are labor-intensive and time-consuming. In addition, their evolvements often require concept enrichment that must be manually reviewed by domain experts. Thus, automatic mechanisms are desirable in both ontology construction and ontology maintenance tasks.

In recent years, many ontology learning (OL) efforts have been made to automate the construction of ontologies from free text [2]. An important subtask in the OL process is relation extraction that aims to extract a novel relationship between known concepts [3]. Putting aside the accuracy of extraction, the discovery of semantic relations from text has its drawbacks: one is that the representations of concepts and relations in the text are usually nonstandard, and the other is that the knowledge extracted from text is often limited and not curated. Due to the widespread use of biomedical ontologies [4], their quality has become very important [5]. As such, in this study, instead of discovering semantic relations from extrinsic information, we investigate an automatic way of uncovering relations between ontology concept names by leveraging the intrinsic knowledge of the ontology itself.

An important observation of biomedical ontologies is that the lexical patterns of the concepts often indicate, to a certain degree, the structural relations between them, especially for hierarchical relations. For instance, in the Foundational Model of Anatomy (FMA) [6], *Left hemidiaphragm* is part of *Diaphragm*, and *Superior mediastinal lymph node* is a *Mediastinal lymph node*. We can notice that in each example, the parent concept name is a substring of the child concept name, as the parent is semantically more general than the child. Using naming conventions in biomedical ontologies is a principle recommended by the Open Biological and Biomedical Ontology (OBO) Foundry [7]. In the literature, lexical-structural relevance had been leveraged for many ontology-related tasks. For instance, we used subphrases of concept names and structural information for disambiguating terms in the FMA [8]. Also, the approach of combining lexical and structural methods is widely adopted in many ontology auditing studies [9-11]. Note that in this paper, we use the terms “concept name” and “term” interchangeably.

In this study, we propose an automatic approach for structuring a given set of concept names based on their lexical granularity. We started by investigating an automatic way to predict the direct relation between 2 given concepts by employing machine learning (ML) algorithms. Since word embedding tools such as Word2Vec [12] and Bert-as-service [13] can extract the semantic features of words and encode the words into feature vectors, relations between words are retained to some extent. By feeding encoded term pairs along with their corresponding relations into ML models such as Convolutional Neural Networks (CNN) [14], Long Short-term Memory Networks (LSTM) [15], or Support Vector Machine (SVM) [16], we can train the models as classifiers to predict the relations between given concept names.

We selected the most common hierarchical relations in biomedical ontologies for experiments: the *is-a* relation and the *part-of* relation. The training dataset comprised randomly selected pairs from the taxonomy and partonomy of the ontologies. Each pair was either directly related by *is-a* or by *part-of*. In addition, we added a third type of concept pairs to the training set: concept pairs that are not directly related (*ndr*). For each pair in the training set, we encoded the 2 terms to vectors using Bert-as-service [13] at first. The subtraction of the 2 vectors formed an input instance for ML models. After training, the models were able to classify a given term pair (A, B) into one of the 3 classes: (A *is-a* B), (A *part-of* B), or (A *ndr* B).

Moving forward, provided with a group of concept names, we aimed to determine how to structure them automatically by utilizing the above ML classifiers. Intuitively, the relative positions of all the concepts can be achieved by pairwise comparisons. However, pairwise comparisons will not only increase the algorithm complexity but also tend to introduce false-positive relations. To deal with this problem, we deployed our previous work [11] on concept granularity to obtain the positions of concepts: Firstly, we determined all the parallel concept sets (PCSs) in the given names. Secondly, we placed them into different hierarchical levels based on their granularity, forming PCS threads. Each thread determined a PCS hierarchy. Lastly, we used the above ML models to determine the relations between neighboring terms along the threads as well as relations between certain terms from different threads. As a result, we achieved the goal of predicting the whole taxonomy and partonomy structures for the given names. To the best of our knowledge, this is the first study that investigates automatic semantic structure generation for a group of concept names in biomedical ontologies.

### Related Work

In the literature, automatic methods were proposed to alleviate human efforts from different aspects of the ontology lifecycle. Many researchers utilized automatic methods to facilitate semantic knowledge extraction for ontology enrichment. For example, Pembeci et al [17] proposed a supervised ontology enrichment algorithm by using concept similarity scores computed via Word2Vec models to discover other related concepts for a given concept. We refer to Liu et al [18] for more references. For ontology concept name prediction, Zheng et al [19] explored deep learning-based approaches to automatically suggest new concept names in the Systematized Nomenclature of Medicine-Clinical Terms (SNOMED CT), under the condition that a bag of words is given. However, only a few studies worked on automating relation prediction and concept organization within ontologies. Zheng et al [20] verified whether an *is-a* link should exist between a new child concept and an existing parent concept in the SNOMED CT. Liu et al [21] proposed a CNN-based method to support the insertion of new concepts into the SNOMED CT: The CNN classifier was trained by vectors translated from concepts using the Doc2Vec algorithm. Afterward, it was able to decide if a given concept has the *is-a* relation with existing concepts in the ontology. Later, they also used a transfer learning method based on BERT to support the insertion of new concepts [22]. A limitation of the work is that at least one parent had to be given for the concept to be inserted beforehand.

Our study differs from the above work mainly in the following aspects: (1) Instead of predicting the insertion place of a new concept or predicting the relation between a particular concept pair, we predict the whole hierarchical structure for a given set of concept names; (2) aside from names of the concepts, we do not need extra information to predict their positions in the whole group; and (3) instead of concatenating the child and the parent, we encode them separately and use their subtraction as an input instance for the ML models.

## Methods

### Materials

We tested our methodology in the FMA [6], which is both a theory of human anatomy and an ontology artifact. In particular, it is a representation of the canonical, phenotypic structure of the human body and its typical components at all biological levels. It is a model suitable for machine manipulation with more than 100,000 concepts, including macroscopic, microscopic, and subcellular canonical anatomy.

For our analysis, we used version 5.0.0 of the FMA (Structural Informatics Group at the University of Washington) [23]. It is distributed as Web Ontology Language (OWL) files, which enables the FMA to be stored in resource-description-frame (RDF) data stores and made available for querying via SPARQL [24]. In this study, we used Virtuoso (version 7.2.5.1; OpenLink Software) as our RDF store [25].

### Model Training and Testing for Direct Relation Prediction

#### Data Preparation

We use the FMA to describe the data preparation process without a loss of generality. We first extracted all the concept pairs directly related by *is-a* or *part-of* from the FMA. The resulting set, *D*, contained 104,665 *is-a* pairs and 61,878 *part-of* pairs. All the children from *D* comprised a set *C*, and all the parents from *D* comprised a set *P*. We then generated the third type of pairs, which were pairs that are not directly related (*ndr*).

The *ndr* pairs consisted of 2 kinds: (1) pairs of terms that share the same ancestor, and (2) pairs comprising a random concept A in the FMA and a child of the sibling of A (ie, uncle-nephew pairs). For the first kind of pair (pairs of terms that share the same ancestor), we first found all the subtrees in the FMA with sizes between 60 and 135. Then, for each of these trees, we let all of its node terms pair with each other. If a direct *is-a* or *part-of* relation did not connect the 2 elements of each pair, it was an *ndr* pair added to the dataset *D*.

The reason that we chose these 2 kinds of *ndr* pairs are the following: Since our ultimate goal was to organize a group of closely related terms, *ndr* pairs in the training dataset should not just be chosen at random. Thus, we intentionally included *ndr* pairs that originated from the same subtrees into the dataset, as the first kind of *ndr* pairs do, to help recognize *ndr* relations in the target groups. As the subtrees should be neither too large nor too small, only subtrees with moderate sizes between 60 and 135 were selected for our experiment. Note that although *is-a* and *part-of* are both transitive relations, indirect *is-a* pairs and indirect *part-of* pairs were classified as *ndr* pairs. For the second kind of *ndr* pairs, we included certain uncle-nephew pairs from the whole FMA dataset, as they tend to be mispredicted to have parent-child relations.

**Figure 1:**
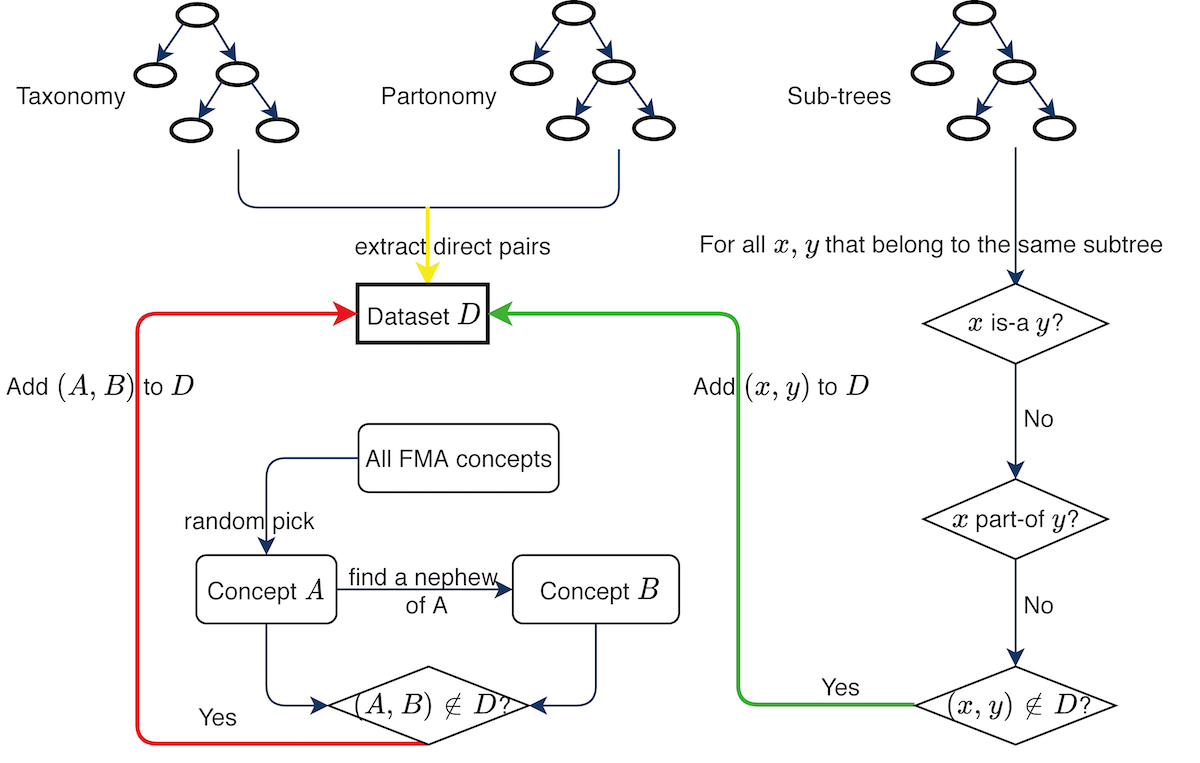
The data preparation process. The final dataset D consists of 3 parts: (1) all of the direct *is-a* and *part-of* pairs in the Foundational Model of Anatomy (inputted by the yellow arrow); (2) *ndr* pairs of terms that share the same ancestor (inputted by the green arrow); and (3) *ndr* uncle-nephew pairs (inputted by the red arrow).

The data preparation process is illustrated in Figure 1. Our selection process of *ndr* pairs stopped when the number of *ndr* pairs reached 3 times the summation of the numbers of *is-a* pairs and *part-of* pairs. The ratio of these 2 kinds of *ndr* pairs was 1:1; that is, we randomly selected 249,815 pairs from the first kind of *ndr* pairs and the same number from millions of uncle-nephew pairs. The number of *ndr* pairs was set much larger than the numbers of *is-a* pairs and *part-of* pairs to better match the real situations in the ontology.

#### Embedding

Our aim was to train an ML algorithm that was able to determine whether 2 given ordered terms maintain 1 of the 3 relations between them, namely, an *is-a* relation, a *part-of* relation, or an *ndr* relation. Above all, the term pairs needed to be converted to vectors, which is called embedding. To do this, firstly, we used the Bert-as-service tool [13] to acquire the vector representations for all words that appeared in the dataset *D*. Each word was represented by a 768-length vector; thus, each concept name in *D* was represented by a sequence of vectors. Secondly, to align all the concept names, we padded all the sequences’ vectors to the same length of 20. Lastly, all the child vectors were subtracted from their respective parent vectors to create the input vectors for classification algorithms. We selected subtraction rather than concatenation because subtraction would catch the differentiation between the parent and the child. As a result, each input vector took shape (1,20,768) and was labeled by its corresponding relation.

#### Model Training and Direct Relation Prediction

We shuffled the input vectors along with their labels and used 80% of them as the training set, 10% of them as the validation set, and the remaining 10% as the testing set. Since FMA terms are all short texts, we selected the classic TextCNN proposed by Yoon Kim [26], which is widely used in short-text classification like our CNN model. The other classification model we used was Bidirectional Long Short-term Memory Networks (Bi-LSTM) [15], which is often used to model contextual information in natural language processing tasks. In our experiments, the parent term and the child term were used as contextual information for Bi-LSTM to predict the relationship between them.

We ran the models using Keras [27] on CentOS with 240 GB of memory and 4 Tesla M60.

In the CNN model, we used 3 Conv1D layers and 2 MaxPooling1D layers following the input layer. After flattening the last layer’s output, we added 2 dense layers such that the former had a *relu* activation and the latter had a *softmax* activation. The cost function we leveraged was *categorical-crossentropy* in Keras. After training, for each input vector that represents a pair of concept names, the CNN model would predict a relation between the 2 concepts.

The second classification model we used was Bi-LSTM. After the input layer, we added a Bi-LSTM layer with 32 memory units in the middle. Then, we flattened the output of the last layer to add a dense layer which had a *softmax* activation. Cost function *categorical- crossentropy* in Keras was also used for classification.

For both models, we set the training data to batches of size 512 and set the *epoch* parameter as 50. For each iteration, we used the validation data to evaluate the model’s performance.

The testing set was used to evaluate the performance of each model. By comparing the predicted results with the real situations in the FMA, we calculated metrics such as the precision, recall, and F1 scores for each model separately.

To demonstrate the robustness of our trained models, we repeated the above experiment 100 times and obtained the average precision, recall, and F1 values. The training set, validation set, and testing set were randomly divided each time, but the 8:1:1 ratio was maintained. In the following step, we selected a particular group of terms from each testing set and automatically obtained the taxonomy and partonomy structures among those terms.

### Automatic Structuring for a Group of Concept Names

#### Algorithm Overview

The above ML models only predict if 2 given terms are directly related by *is-a* or *part-of*. However, rather than predicting the relation between 2 random terms, a more meaningful use lies in the organization of a given set of closely related terms. To achieve this goal, we needed to obtain their relative positions.

An intuitive solution is to use the pairwise comparison. Let the target term set be Q. Suppose the number of terms in Q is *M*; we will need *M*(*M−1*) times of testing to obtain the pairwise relations among them. However, apart from time complexity, another problem with this solution is that it will introduce too many *ndr* pairs since real *is-a* or *part-of* relations in Q are quite sparse. Thus, the prediction results for *ndr* pairs will easily affect the prediction results for *is-a* and *part-of* pairs.

**Figure 2:**
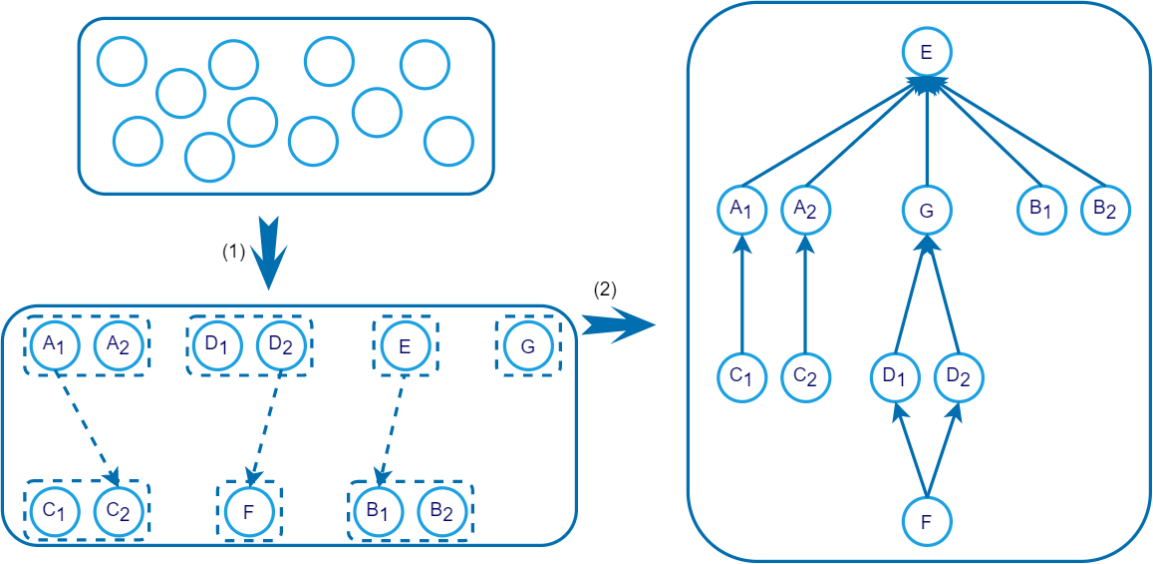
The use of lexical granularity to obtain the relative positions of terms. (1) Parallel concept sets (PCS) and PCS thread detection; 7 PCS nodes and 4 PCS threads were detected in this example. PCS: represented by dashed rectangles; Concept names: represented by circles; Substring relations: represented by dashed arrows. (2) Relation prediction. *is-a* or *part-of* relations predicted by the classification model: represented by solid arrows.

As such, to reduce the use of pairwise comparison, we deployed our previous work [11] on concept granularity to obtain the relative positions of the terms. Specifically, we divided the target set into small parallel concept sets (PCSs) [11]. As parallel concepts remain at the same level of granularity, it turned out that, in the end, we only needed to organize the PCSs. To do this, we first placed the PCSs into different hierarchical levels based on their granularity, forming PCS threads. Each thread determined a semantic hierarchy. Then, we determined the hierarchy between different threads. Then, after the whole structure was obtained, we utilized the trained ML models to predict the relations between directly connected terms and thus obtained the whole semantic map. This procedure is briefly illustrated in Figure 2.

#### PCS Detection

A parallel concept set (PCS) is a set comprised of concepts sharing the same level of conceptual knowledge [11], such as symmetric concepts. A pair of concepts is called symmetric if the concept names are the same but for the possible difference

in a single occurrence of the modifiers used [10]. For instance, *Lower extremity part*-*Upper extremity part* is a symmetric concept pair concerning the symmetric modifier pair *Upper* and *Lower*.

In order to detect all the symmetric concept pairs in *Q*, we needed to retrieve all the symmetric modifier pairs first. To do this, we used the Stanford Parser [28] to obtain all the noun-phrase (NP) chunks without prepositions. For all the modifiers in those chunks, any 2 of them that share a common context were selected to form a modifier pair. After retrieving all the symmetric modifier pairs from *Q*, we easily detected all the symmetric concepts using SPARQL queries [24]. In the end, every symmetric term pair formed a PCS. For terms whose symmetric counterparts could not be found in *Q*, each of these formed a PCS by itself.

#### PCS Thread Detection

As noted, for hierarchical relations, the parent term is more general than the child term and is usually a substring of the child term. As a result, we can leverage the substring threads in Q to organize the PCSs identified from the above step. We used *A* 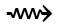 *B* to represent that *A* is a substring of *B*. A substring thread *A_0_* 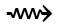 *A_1_*...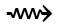...*A_n−1_* 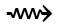 *A_n_* would correspond to a parent-child thread *A_0_* 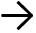
*A_1_*...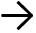..*A_n−1_*
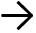
*A_n_*. Along each parent-child thread, we generalized every term node to the PCS that the term belonged to. As a result, all the PCSs were organized into several threads. Each thread was named a PCS thread. Note that some threads may only contain 1 PCS node. Also, some PCSs may appear in several threads.

#### Relation Prediction

**Figure 3:**
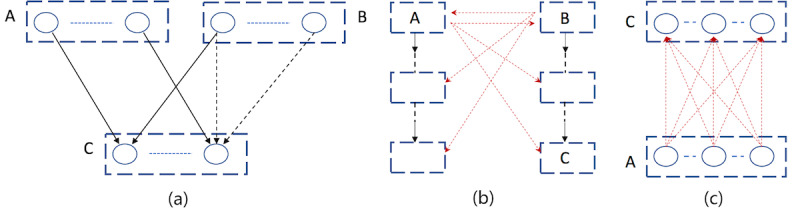
Determination of term pairs to be fed into machine learning models for relation prediction. Parallel concept sets (PCSs): represented by rectangles; Concept names: represented by ovals. (a) A, B, and C are 3 PCS nodes; A, C and B, C are neighboring nodes along 2 PCS threads, respectively. Substring relations: represented by solid arrows. As the right-most term C has no substring term in B, it is paired with every term in B, represented by dashed arrows. Each arrow (solid or dashed) connects 2 terms such that the relation between them is predicted using classification models. (b) A and B are 2 different PCS thread roots. Each root is paired with every PCS node in other threads under different roots; red dashed arrows are used to connect them. For instance, (C, A) is such a pair. (c) Classification models are used to predict the pairwise relations between concept names in C and A from the above step.

The relative positions of nonroot PCS nodes were determined. Hence, we no longer looked for their parents elsewhere but only predicted relations between concepts in neighboring nodes. Specifically, we first paired each term in the PCS with its substring term in the parent PCS. If no substring term was found in the parent PCS, the term would be paired with every item in the parent. As illustrated in Figure 3a, A, C, and B, C were neighboring nodes along 2 PCS threads, respectively. Since the right-most term in C had no substring in B, it was paired with every term in B, represented by dashed arrows. Then, we predicted the relations between the paired terms by leveraging the previously trained classification models.

In regard to the PCS thread roots, if all the threads shared 1 root, no further treatment was needed. If there existed more than 1 different thread root, we still leveraged pairwise comparison to determine the parents for the roots: For each PCS thread root, we first paired it with every PCS node in other threads, as illustrated in Figure 3b. For instance, (C, A) was such a pair, with C as the parent PCS and A as the child PCS (Figure 3c). We used the ML models to predict the pairwise relations between terms in those paired PCS nodes. As Figure 3c illustrates, each term in C was paired with every term in A, and the specific relation between each pair would be predicted by the previously trained classification models.

Lastly, only *is-a* and *part-of* edges would be retained. For the given group of terms, suppose the number of predicted *is-a* relations was *P*, and the number of correct ones among them was *CP*; then, the precision for *is-a* was calculated as *CP/P*. Further, suppose the number of original *is-a* relations in the FMA was *O*, and the number of them that were correctly predicted was *CO*; then, the recall for *is-a* was *CO/O*.

### Case Studies

To test the generalizability of our method, we selected a group of terms from each of the testing sets in the 100 cross-validation experiments for automatic structuring. As mentioned, the most useful scenario happens when the terms are closely related instead of semantically distant. Thus, we only selected terms that belong to the same tree for experiments.

The process was as follows: Firstly, we collected all the term roots in the testing set and collected all the *is-a* and *part-of* descendants under them, forming a concept tree for each root. Secondly, we picked out the trees with more than 20 elements. Lastly, we randomly selected a tree and created a set formed by all the terms in that tree as our study case. Note that we manually assured that none of the concepts in the case studies had appeared in the training set.

For the selected 100 cases, we followed the steps described above to predict the whole semantic map among the concept names in the groups. Our experiments separately leveraged the 2 previously trained models for direct relation prediction. By comparing the predicted results with the real cases in the FMA, we evaluated the performance of our methodology by calculating the average precision, recall, and F1 values for all the cases.

For a more specific analysis of the results, we selected the largest case with root “First Rib” among the 100 cases. The set contained 57 concepts with 89 relations among them in the FMA, including 34 *is-a* relations and 55 *part-of* relations, as shown in Multimedia Appendix 1.

To demonstrate the advantage of our PCS-based method, we performed another group of experiments for the case study on “First Rib” based on primitive pairwise comparisons among the whole set of concept names. We fed 3192 (from 57×56) term pairs to the models for direct relation predictions. Then, a comparison between the PCS thread-based method and the primitive pairwise-based method was made for this case.

## Results

### Classifiers Can Predict the Direct Relation Between 2 Given Concept Names

Using the remaining 10% of the data as the testing set in each of the cross-validation experiments, we evaluated the performances of the 2 models on direct relation prediction between 2 given concept names. The average results are shown in Table 1. The table shows that the models performed well on this task, with both precision and recall above 0.9. This demonstrates that machine learning models, when trained by existing relations in the ontology, can be very effective at predicting relations between new incoming concepts, provided that their names are given. Based on this result, we invented the PCS thread-based method and further investigated the possibility of organizing a group of terms.

**Table 1:** Average performances of the 2 models on direct relation prediction (100 rounds).

Model	*is-a*		*part-of*		*ndr*		Overall		
	P^a^	R^b^	P	R	P	R	P	R	F1
Bi-LSTM^c^	0.93	0.91	0.90	0.91	0.97	0.93	0.95	0.92	0.93
CNN^d^	0.91	0.90	0.89	0.90	0.94	0.92	0.92	0.91	0.91

^a^P: precision.

^b^R: recall.

^c^Bi-LSTM: Bidirectional Long Short-term Memory Networks.

^d^CNN: Convolutional Neural Networks.

### Automatic Structuring of Groups of Closely Related Terms

In the 100 testing sets, we found that the sizes of all trees were less than 60, and the 100 term groups we selected had an average size of 25. The smallest group contained 20 terms and the largest group contained 57 terms.

We applied the PCS-based algorithm to the 100 cases and calculated the average precision, recall, and F1 values for *is-a* and *part-of* based on Bi-LSTM and CNN, respectively. The results are shown in Table 2; the overall F1 score using Bi-LSTM was 0.79, which slightly outperformed the algorithm using CNN.

**Table 2:** Average performances of the parallel concept set (PCS) thread-based algorithm on 100-term groups.

Model	*is-a*		*part-of*		Overall		
	P^a^	R^b^	P	R	P	R	F1
Bi-LSTM^c^	0.84	0.79	0.82	0.68	0.83	0.76	0.79
CNN^d^	0.72	0.79	0.72	0.69	0.72	0.76	0.74

^a^P: precision.

^b^R: recall.

^c^Bi-LSTM: Bidirectional Long Short-term Memory Networks.

^d^CNN: Convolutional Neural Networks.

**Figure 4:**
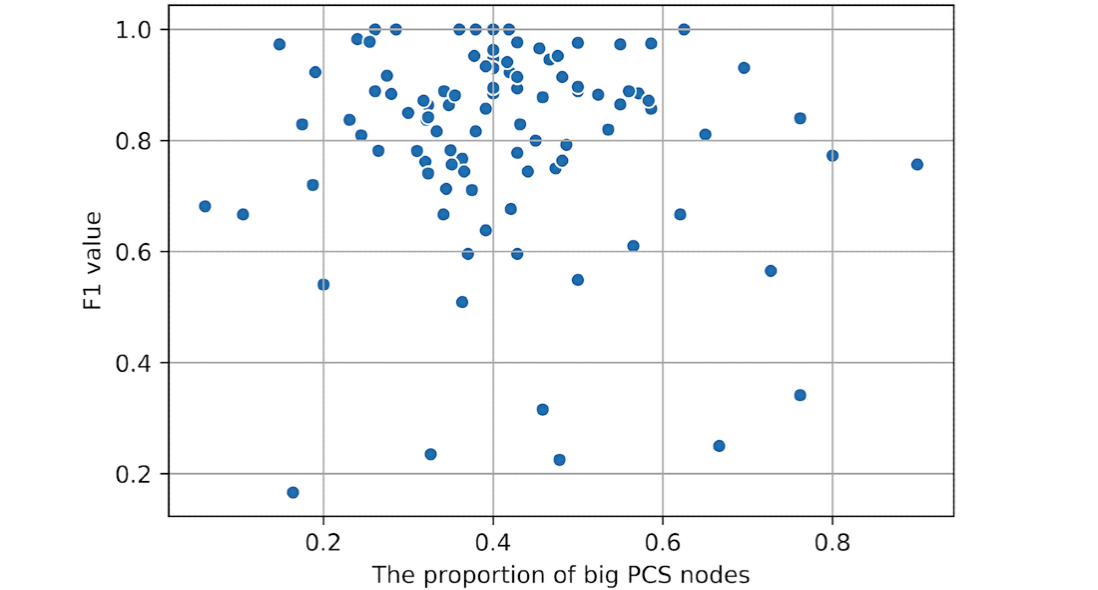
The relation between the proportion of big parallel concept set (PCS) nodes and the F1 value for 100 cases.

To analyze the influence of PCS nodes that contain at least 2 symmetric terms (ie, big PCS nodes) on the performances of the above algorithm, we calculated the proportion of big PCSs among all the PCSs for each study case and demonstrated the relation between the proportion and the F1 value (Figure 4). As it indicates, the PCS-based algorithm's performance does not have evident relevance with the richness of big PCS nodes.

Further, to demonstrate the usefulness of ML models in our approach, we collected all the *is-a* and *part-of* pairs without substring relationships in the 100 study cases and found 652 such pairs. Among the 652 pairs, 235 (36%) could be correctly predicted by our algorithm using both models. For instance, we correctly predicted the relation (*Endplate of intervertebral disk,* *is-a*, *Organ component)* in which the 2 terms have no shared word. In fact, the above ratio could have been much higher if the pairs had actually fed into the ML models, as some pairs without substring relationships were filtered out by the algorithm beforehand. On the other hand, the 100 cases contained 1140 *ndr* pairs in which 1 term is a substring of the other. Of the 1140 *ndr* pairs, 931 (82%) were correctly predicted by both models as *ndr* pairs.

As the above results show, our proposed algorithm works well on both term pairs, with or without obvious lexical patterns.

### Specific Case Study on “First Rib”

For the specific case on “First Rib,” in the 57 concept names to be structured, we detected 1 symmetric modifier pair, (*left*, *right*). We first divided all the concepts into 37 PCSs. Of these 37 PCSs, 20 PCSs contained 2 terms and 17 PCSs contained only 1 term. Then, based on lexical granularity, we found 29 PCS threads. Except for 1 thread that took “Fossa for first costal cartilage” as its root, all the other 28 threads shared the same root: “First Rib.”

The results of the automatic structuring of term groups based on PCS threads and pairwise comparisons are shown in Table 3. The PCS thread-based method has higher precision, and the pairwise-based method has higher recalls. The reason is that the PCS thread-based method had filtered out certain pairs, including those with real *is-a* or *part-of* relations between their elements. On the other hand, the introduction of false-positive results was expected of the pairwise method.

We analyzed the results from the “First Rib” case for our PCS thread-based algorithm concerning the Bi-LSTM model to demonstrate why some relations were wrongly predicted or missed.

**Table 3:** The parallel concept set (PCS) thread-based algorithm versus the primitive pairwise-based algorithm on the “First Rib” case, using different models.

Model and Algorithm		*is-a*		*part-of*		Overall		
		Precision	Recall	Precision	Recall	Precision	Recall	F1
**Bi-LSTM^a^**								
	Alg_1_^b^	1.0	1.0	0.71	0.63	0.83	0.78	0.80
	Alg_2_^c^	0.94	1.0	0.55	0.90	0.66	0.94	0.78
**CNN^d^**								
	Alg_1_	0.97	1.0	0.73	0.64	0.83	0.78	0.80
	Alg_2_	0.58	1.0	0.42	0.98	0.47	0.98	0.64

^a^Bi-LSTM: Bidirectional Long Short-term Memory Networks.

^b^Alg_1_: PCS thread-based algorithm.

^c^Alg_2_: pairwise-based algorithm.

^d^CNN: Convolutional Neural Networks.

### Result Analysis on “First Rib” for the PCS Thread-based Algorithm Concerning The Bi-LSTM Model

Using the Bi-LSTM model, our approach predicted 83 relations among the group of concept names, including 34 *is-a* relations and 49 *part-of* relations. The results are illustrated in Multimedia Appendix 2. All of the 34 *is-a* relations in the FMA were successfully discovered by the model.

Compared to the 55 real *part-of* relations in the FMA, 35 *part-of* relations were correctly predicted, which means that 20 *part-of* relations in the FMA were missed by our method and 14 predicted *part-of* relations were unexpected. As a result, we achieved an overall precision of 0.83 [from (34+35)/83] and a recall of 0.78 [from (34+35)/89], as shown in Table 3.

The 14 unexpected *part-of* relations that do not exist in the FMA can be divided into 3 types: (1) detected *part-of* relations that connected the child to a further parent than that of the FMA; (2) detected *part-of* relations that connected the child to a closer parent than that of the FMA; (3) detected *part-of* relations that did not exist in the FMA and had no counterpart relations in the FMA.

**Figure 5:**
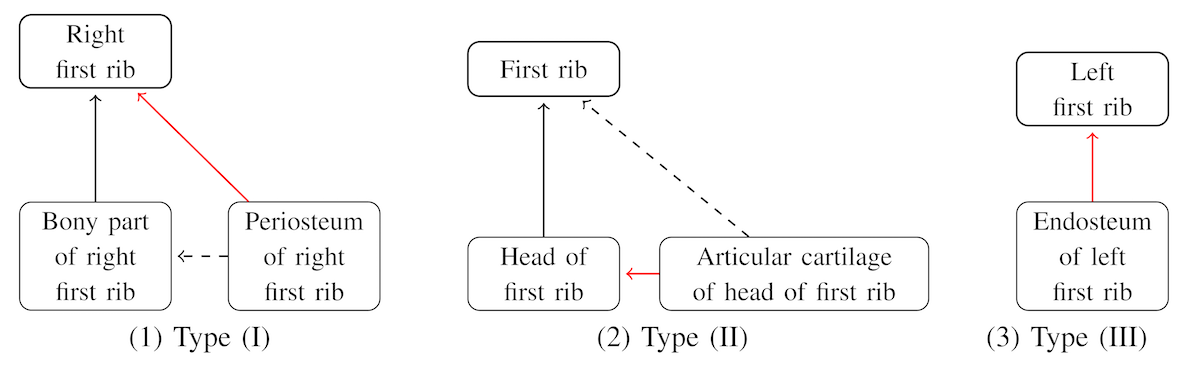
Types of unexpected *part-of* relations that do not exist in the Foundational Model of Anatomy (FMA). Black solid arrows represent relations in the FMA successfully predicted by the model. Red arrows represent relations predicted by the model but not in FMA. Dashed arrows represent relations in FMA that were missed by the model. (1) Example of the first type of predicted relations; (2) example of the second type of predicted relations; (3) example of the third type of predicted relations.

The first type was detected *part-of* relations that connected the child to a further parent than that of the FMA. As illustrated in Figure 5, Type I, *Periosteum of right first rib* has a closer parent, *Bony part of right first rib*, in the FMA, but the algorithm connected it to its ancestor, *Right first rib*. The reason is that the node *Bony part of first rib* is not lexically a substring of *Periosteum of right first rib* and thus did not appear in the corresponding PCS thread. Six predicted relations took this type.

The second type was detected *part-of* relations that connected the child to a closer parent than that of the FMA. As illustrated in Figure 5, Type II, the algorithm predicted the parent of *Articular cartilage of head of first rib* to be *Head of first rib*” instead of *First rib*. The reason is that only relations between neighboring terms along PCS threads would be predicted, but *Head of first rib* is in the middle of the other 2 terms. Six predicted relations took this type.

The third type was detected *part-of* relations that did not exist in the FMA and had no counterpart relations in the FMA. However, the parent term is a substring of the child term, as illustrated by the example in Figure 5, Type III. Two predicted relations took this type.

Although the above instances do not exist in the FMA, they are not all semantically wrong. For example, instances of the first type can be inferred from relation transitivity. Moreover, compared to the real cases in the FMA, the 6 instances of the second type were more reasonable because they show a finer granularity than their counterparts in the FMA. Also, the 2 instances of the third type were semantically correct.

On the other hand, the 20 missed *part-of* relations happened due to 1 reason: their parent-child term pairs were not fed to the model for prediction. As already described, for terms in nonroot PCS nodes, we only searched for their parents in neighboring parent PCSs. For the 20 missed cases, the parent and the child were not in neighboring PCSs and thus could not be discovered by our algorithm. For instance, in Figure 5, Type I, *Bony part of right first rib* and *Periosteum of right first rib* were not in neighboring PCS nodes along any thread, and thus, the pair was not fed to the model for relation prediction. Amongst the 20 missed cases, 11 cases came along with the instances of the first and second types of unexpected *part-of* pairs that did not exist in the FMA. As illustrated in Figure 5, Types I and II, while the model predicted an extra new relation, it would miss an old relation (a red arrow co-occurred with a dashed arrow). Only the first type of instance did not appear in the missed case because the intermediate parent was not in the term group.

If those missed parent-child term pairs were fed into the Bi-LSTM model, could they be correctly detected? Table 3 shows that the recall for Bi-LSTM was 0.94, which means that most of the original relations in the FMA could be successfully detected by the model if fed for prediction. However, as seen in the results, the precision values would drop greatly for primitive pairwise comparisons.

If the group of concept names to be structured do not show much relevance in their linguistic features, the number of PCS thread roots will increase. Under that circumstance, as our algorithm pairs each root with every term in the other threads (Figure 3b), the number of term pairs fed to the ML models for relation prediction will increase. In the extreme case, all of the terms are roots by themselves, and the algorithm will turn into a pure pairwise-based algorithm. Fortunately, biomedical ontologies follow certain naming conventions, and meaningful usage of our methodology lies in the construction of a group of terms that are semantically close to each other; however, PCSs will play an important role in most cases.

## Discussion

### Principal Findings

This study proposes an innovative approach to the automatic construction of a given set of concept names with regard to *is-a* and *part-of* relations, which can save significant labor for domain experts in the ontology construction process. Our method comprises 2 main steps: (1) automatic prediction of direct semantic relation between 2 concept names using classification models; experiments on the FMA show that machine learning models can predict if 2 new terms are directly related by a *is-a* or *part-of* relation, provided that they are trained by existing relations; and (2) automatic construction of a group of closely related concept names based on PCS threads. First, we detected all the PCSs in the group and organized them into PCS threads based on lexical granularity. Second, we obtained the relative positions of different threads and the whole structure of the group. Lastly, we determined whether there exists an *is-a* relation or a *part-of* relation between each directly connected term pairs, thus completing the construction of the taxonomy and the partonomy structures.

Some concepts may have multiple *is-a* or *part-of* parents. As analyzed, for terms in nonroot PCS nodes, except for the threads they belong to, we do not look for their parents in other threads anymore. However, since PCS threads may have convergences, some terms may still be predicted to have multiple parents. In fact, no matter whether it is for *is-a* or *part-of*, parents that are not substrings of nonroot PCS nodes will be overlooked by our algorithm, such as the missed *part-of* instances in the FMA. On the other hand, all of the *is-a* relations were successfully discovered because all of the *is-a* parents appeared above their children along certain threads.

It is not a simple transition from step 1 to step 2. As shown by our results, even though the performances of ML models on relation prediction for randomly selected pairs may be quite promising (Table 2), it was still difficult to obtain the semantic structure for a set of terms using pure pairwise comparisons (Table 3). The reason is that pairwise comparisons introduce too many pairs: *N* terms will generate *N*(*N*−1) pairs since direction matters. As the real connections among those terms can be quite sparse, most of the pairs are actually *ndr* pairs, which tremendously exceeds *is-a* pairs and *part-of* pairs. As such, even a small portion of *ndr* relations that were wrongly predicted as *is-a* or *part-of* relations could greatly decrease the precision of the results for *is-a* and *part-of* relations. That is why we introduced the PCS-based method, which only tested pairs that have a high possibility of exhibiting *is-a* or *part-of* relations between their elements. As a result, the number of false *is-a* relations and false *part-of* relations was reduced. However, on the other hand, the reduction of term pairs in the PCS-based stage has its drawback. As the step filters out many *ndr* pairs, it also misses some real relations between terms that are not lexically related, which is why the recall values for the PCS-based method were lower than that of the pairwise-based method.

### Future Work

To improve the performance of our PCS-based method, we need to include more possible pairs to be inputted into the ML models for relation prediction. This requires a mechanism to be able to identify hierarchical relations between terms that are not lexically related, and in the meantime, to avoid introducing false-positive results. The difficulty lies in the ability to distinguish the *ndr* pairs from the other 2 relations. Future research may focus on the following aspects: (1) Although we enlarged the set of *ndr* pairs in this study, it is still impractical to collect all possible *ndr* pairs for classification; we will try ML algorithms that are able to classify the relations based only on positive samples, and hence, there will be no need to collect *ndr* pairs then. (2) Except for the lexical information of the concept names, we will try including additional knowledge such as metadata or even structural information to the embedding framework. (3) We tried 2 classic ML models in this study and did not apply too much effort to parameter tuning or model refinements. We believe that further exploration of this aspect will also help.

Also, to make the methodology provided in this study scalable to more cases in diverse ontologies such as SNOMED CT [29], the key is for the ML models to be able to “interpret” the semantic meaning behind each biomedical term. This will require a suitable embedding method in the biomedical field. In the future, we will try other embedding methods learned from multiple sources of biomedical data, such as Cui2Vec [30] or BioBert [31], to generalize the method to other cases.

The 100 cases we experimented with in the FMA are not large because the closely related term trees in the testing sets are relatively small. If the target group is much larger, the performance of the proposed algorithm may not be as strong since more terms will increase the number of *ndr* pairs. In the future, we will try the methods mentioned above and will work on larger term groups.

This study is an initial step toward automated ontology construction. As the training dataset is collected from the same ontology, the methodology we proposed in this study is applicable, provided that a part of the ontology is already known. To structure an ontology from scratch, the relations between entities will have to be learned from other knowledge sources such as the UMLS [32] or the literature. We believe our study will provide insight for future studies in this field. Moreover, the methodology provided here can be easily deployed for determining insertion positions for incoming concepts in ontology enrichment processes. Also, as the results show, some predicted relations are more reasonable than the real cases, which indicates that ontology quality assurance tasks can also benefit from this study.

### Conclusions

In this study, given a set of closely related concept names in the FMA, we investigated an automatic way to generate the taxonomy and partonomy structures for them. We trained machine learning models to predict if there exists a direct hierarchical relation between 2 given terms; based on this, we further proposed an innovative granularity-based method to automatically organize a given set of terms. The 100 cases that we studied in the FMA demonstrated that our method is effective for structuring ontology concepts automatically, provided that their names are given. We believe this pioneering study will shed light on future studies on automatic ontology creation and ontology maintenance.

## Appendix

Multimedia Appendix 1 The original hierarchy map for the concept group “First Rib” in the Foundational Model of Anatomy. Red arrows represent 34 *is-a* relations; gray arrows represent 55 *part-of* relations.

Multimedia Appendix 2 Result for the automatic structuring of 57 concept names. The nodes represent the 37 parallel concept sets (PCSs); dashed rectangles represent PCSs with more than 1 term. The nodes in green are the 2 thread roots; arrows connect terms instead of PCSs. Gray arrows represent the 69 correctly predicted *is-a* and *part-of* relations; yellow arrows represent the 20 missed *part-of* relations; red arrows represent the 14 predicted *part-of* relations that do not exist in the Foundational Model of Anatomy (FMA).

## Abbreviations

Bi-LSTM: Bidirectional Long Short-Term Memory Network
CNN: Convolutional Neural Networks
FMA: the Foundational Model of Anatomy
ML: machine learning
OL: ontology learning
OWL: Web Ontology Language
PCS: parallel concept set
RDF: resource description frame
SNOMED CT: systematized nomenclature of medicine-clinical terms
SVM: support vector machine
UMLS: Unified Medical Language System
