# Supplementary figures and images for: Automatic Structuring of Ontology Terms Based on Lexical Granularity and Machine Learning: Algorithm Development and Validation
Source: JMIR Med Inform. 2020 Nov 25;8(11):e22333. doi: 10.2196/22333 (PMC7725650; doi:10.2196/22333)

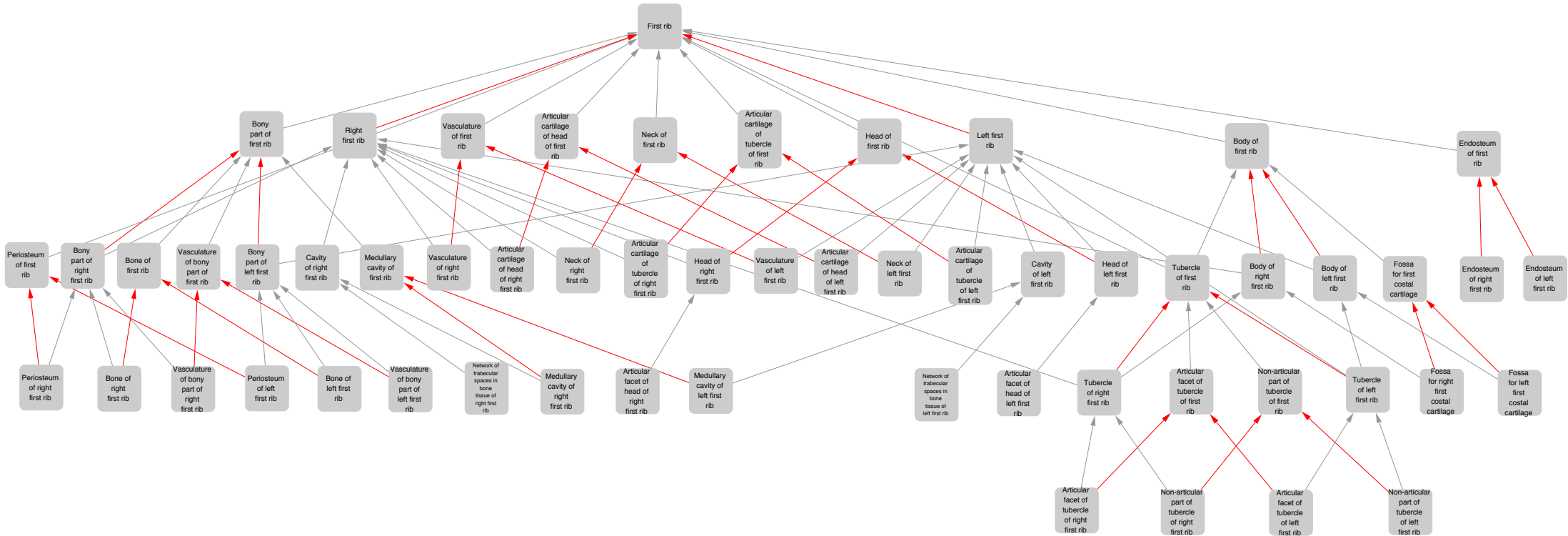

Supplement: Multimedia Appendix 2 [file medinform_v8i11e22333_app2.pdf]
